# Risk of acquiring *Ascaris lumbricoides* infection in an endemically infected rural community in Venezuela

**DOI:** 10.1017/S0950268822001273

**Published:** 2022-07-29

**Authors:** Renzo Nino Incani, Lapo Mughini-Gras, Tobias Homan, Ivan Sequera, Luis Sequera, Ruth Serrano, Carlos Sequera, Luis Salas, Marisabel Salazar, Paola Santos

**Affiliations:** 1 Department of Parasitology, Faculty of Health Sciences, Universidad de Carabobo, Valencia, Venezuela; 2 Faculty of Veterinary Medicine, Institute for Risk Assessment Sciences (IRAS), Utrecht University, Utrecht, The Netherlands; 3 Center for Infectious Diseases Control, National Institute for Public Health and the Environment (RIVM), Bilthoven, The Netherlands; 4 Faculty of Earth and Life Sciences, Vrije Universiteit Amsterdam, Amsterdam, The Netherlands

**Keywords:** *Ascaris lumbricoides*, pyrantel, risk factors, soil-transmitted helminthiasis

## Abstract

Soil-transmitted helminths, such as *Ascaris lumbricoides*, are the most prevalent parasites globally. Optimal anthelmintic treatment for *A. lumbricoides* in endemically infected communities is challenged by several host-related and environmental factors influencing infection acquisition. We assessed the risk of *A. lumbricoides* (re)infection after treatment in a Venezuelan rural community. Individual merthiolate-iodine-formaldehyde-fixed faecal samples were collected from 224 persons before a single-dose pyrantel treatment and at 1, 3, 6, 9 and 15 months after treatment. Effects of age, sex and socioeconomic status (SES) on *A. lumbricoides* prevalence, eggs/gram faeces (EPG) and infection (re)acquisition were assessed using both generalised linear mixed-effects models and survival analysis. Pre-treatment *A. lumbricoides* prevalence was 39.7%. Higher prevalence was associated with younger age and lower SES. Higher EPG values were observed among young children. Median time to *A. lumbricoides* infection was six months after treatment: at 1, 3, 6, 9 and 15 months post-treatment, cumulative incidence was 6.7%, 18.9%, 34.6%, 42.2%, and 52.6%, respectively. Younger age, lower SES, and pre-treatment *A. lumbricoides* infection status showed significantly elevated hazard ratios. Mass drug administration protocols would benefit from considering these factors in selective treatment strategies and possibly more than just annual or biannual treatments in the target population.

## Introduction

Soil-transmitted helminthiases (STH), such as the one caused by *Ascaris lumbricoides*, are the most prevalent parasitic infection globally. Recent estimates from the 2019 Global Burden of Disease Study indicate that around 446 million people worldwide are infected with *A. lumbricoides*, with an estimated disease burden of 754 000 Disability Adjusted Life Years (DALY) [1]. Moreover, 1.5 billion people worldwide are estimated to be affected by STHs, accounting for an estimated global disease burden of 1.5 million DALY [1]. Additionally, 5.9 billion people worldwide are estimated to be at risk of acquiring a STH [2, 3].

*A. lumbricoides* is an anthroponotic intestinal helminth acquired via the faecal-oral route. Adult male and female worms live and copulate in the human intestinal lumen where female worms lay eggs that are released into the environment with the faeces. Unfertilised eggs can also be laid in absence of copulation, but these eggs are not infective. Fertilised eggs need to mature in the environment to embryonate and become infective, which takes from 10 to 50 days, depending on the specific environmental conditions [4]. Infection happens when a human host ingests embryonated eggs through soil-contaminated food or water, as well as through ingestion of eggs on contaminated fomites, body parts (e.g. dirty hands) and the soil itself. Once ingested, eggs hatch in the intestine and the ensuing larvae penetrate the gut wall, migrate via the hepatic-portal blood to the heart and lungs, ascend through the trachea and larynx, and are eventually swallowed back to the intestine where they mature to adults and start laying eggs. This entire pre-patent period usually lasts 9–11 weeks [5].

Anthelmintic treatment is one of the most common methods to reduce STH prevalence at the community level, with preventive chemotherapy based on anthelmintic mass administration to pre-schoolers (24–59 months of age) and school-aged children (5–12 years of age) often used as the primary intervention to reduce morbidity, as advised by the World Health Organization (WHO) [6–9]. However, to interrupt STH transmission, anthelmintic administration to adults seems necessary, for a period of at least ten years and eventually establish complementary interventions, such as provision and usage of WASH (water, sanitation and hygiene) resources [10–14].

Optimal use of anthelmintics against *A. lumbricoides* in endemically infected communities is challenged by several host-related and environmental factors influencing the risk of acquiring *A. lumbricoides* infection after treatment, which vary across cohorts. The type, dosage, coverage, effectiveness and frequency of administration of anthelmintic drugs, as well as several demographic characteristics (e.g. age, gender, occupation, education, financial income, family structure, geographical location, social class, etc.) of the affected individuals and their households, are some of the factors to consider [10, 15–21]. STH control in Venezuela has had a history of success for the last six decades, with *A. lumbricoides* prevalence being reduced from nearly 60% in the 1940s and 1950s to 27% in the early 1990s, and to 5% more recently (2007–2010). The latter prevalence estimate concerns urban areas and might be partly due to the STH control programmes, as well as changing living conditions, whereas in rural areas prevalence can still be as high as 40%. In the last two decades, very limited and irregular actions have been taken to administer anthelmintic drugs in schools [22]. In 2019, an attempt at preventive chemotherapy using mass drug administration was made following WHO recommendations, administering 4 million doses of albendazole to both pre-schoolers and school-aged children in urban and rural areas. Another attempt was performed in 2021 on a comparable number of children, both in rural and urban areas, covering between 60 and 80% of the target population. However, no coprological testing could be performed, neither pre- nor post-treatment, to assess the impact of the intervention. At present, Venezuela is listed by the WHO as having a coverage of mass drug administration among children of 75% or more, for a period of less than five years [8].

Here, we present the results of a longitudinal field study in which we assessed the risk of acquiring *A. lumbricoides* infection after anthelmintic treatment in an endemically infected rural community in Venezuela. This study was performed to assess specifically: (1) how long it takes for *A. lumbricoides* infection levels in the community to build up again after treatment, (2) which groups of people are more likely to acquire the infection, and (3) at which rate they acquire the infection. The results of this study are expected to provide guidance to mass drug administration strategies for ascariasis in this type of resource-limited settings where the prospects of routinely assessing STH control interventions to identify opportunities for improvement are yet to be implemented.

## Methods

### Study community and ethical clearance

The study was conducted in ‘Caserío El 25’, a rural community located in the Carabobo state, Venezuela. A detailed description of this community can be found elsewhere [23]. This study was part of a larger research project aimed at elucidating the epidemiology of intestinal parasite infections in Venezuela's rural communities. The objectives of the project were explained to the members of each household in the study community to obtain written informed consent from all adults and parents or legal guardians of children (<18 years old). The study adhered to local ethical criteria and was approved by the Ethical Committee of the Carabobo State Health Authority (INSALUD), Venezuela, and by the Ethical Committee of the VU University of Amsterdam, the Netherlands. The study design was longitudinal and involved the investigation of factors influencing the risk of *A. lumbricoides* (re)infection. Anthelmintic treatment (pyrantel) was given to the study participants to define the start of the at-risk period for the time to (re)infection. At the time of the study (December 2008–March 2010), the community was composed of 470 inhabitants living in 85 houses. The whole community was invited to participate and 224 people across 55 houses consented and were enrolled into the study.

### Sampling procedures

In total, six consecutive samples of faecal material were obtained from each participant. The first sample was collected pre-treatment (‘baseline’ sampling), and the subsequent five samples were collected at 1, 3, 6, 9 and 15 months post-treatment. Each sampling lasted a maximum of three days, meaning that participants provided the samples within a temporal window of three days.

### Treatment

Pyrantel was administered as a single oral dose with a suspension of 10 mg/kg, two weeks after the baseline sampling. Fourteen participants in the baseline sampling could not be treated because they were absent from home when the treatment was administered. These participants were therefore excluded from further sampling. At the end of the study (i.e. after the sampling at 15 months post-treatment), all participants were offered (free of charge) a single dose of pyrantel as a deworming agent. Pyrantel as a paralyzing anthelminthic drug was used instead of albendazole because this study was part of a larger study that also included adult *A. lumbricoides* worm expulsion.

### Collection and processing of faecal samples

Faecal samples were collected individually in 10 ml faecal collectors, kept refrigerated and transported to the laboratory at the University of Carabobo in Valencia, Venezuela, the same day of collection. At the laboratory, 0.15–0.50 g of each sample was added to a pre-weighed 7 ml rubber stoppered Vacutainer^R^ tube with 4.5 ml merthiolate-iodine-formaldehyde (MIF) fixative in an electronic scale with a sensitivity of 1/10 of a mg. Timing between faeces collection and fixation in the laboratory was between 6 and 8 h. Tubes were then reweighed after adding the faeces to obtain the amount of faeces examined from each participant. After vortexing the tubes for 5 s, 100 μl of faecal suspension was transferred onto microscopy slides, covered with 24 × 50 mm coverslips, and sealed with melted paraffin to avoid desiccation. Two samples of faecal suspension per tube (i.e. per participant) were thoroughly examined at the microscope to count *A. lumbricoides* eggs and to estimate the number of eggs per gram (EPG) of faeces. A third slide was examined when the difference between two counts was more than 20% [24, 25]. While MIF and Kato-Katz techniques show good agreement when two MIF slides are examined [25], MIF was preferred over Kato-Katz because it eliminates the urgency for sample examination that Kato-Katz requires and, therefore, MIF is preferred when processing large numbers of samples in a short period. Additionally, MIF allows for re-examination of samples and easier detection of high parasitic loads, while Kato-Katz egg count at high parasitic loads becomes difficult. Kato-Katz has been used in the same faecal samples to compare its performance with MIF and the results have been reported previously [25].

### Data analysis

Prevalence and mean intensity of infection (i.e. EPG among the positive samples) were calculated at each sampling period. Potential effects of participants' sex and age before treatment (≤5, 6–10, 11–15, 16–25, 26–45, ≥46 years) were assessed over sampling periods. Moreover, previous studies in the same area identified a number of factors associated with STH infection risk referring to socio-economic conditions [23]. Therefore, a variable indicating the socio-economic status (SES) of the households was defined using the Graffar scale [26], which measures SES based on five factors: the occupation of the ‘head of the household’ (i.e. the person with the highest level of occupation), maternal educational attainment, household income, living conditions in the home and state of the neighbourhood of residence. The scale consists of five classes and all households in this study fell within the two lowest classes: lower-middle SES (class IV) and lower SES (class V). Potential effects of SES on *A. lumbricoides* prevalence and intensity of infection were also assessed over sampling periods and statistical analysis was performed using multivariable generalised linear mixed-effects models (GLMMs) with a logit link and binomial error distribution (for dichotomous data, i.e. positive/negative samples), or a log link function and negative binomial error distribution (for count data, i.e. EPGs). The GLMMs included two nested random effects to account for repeated measurements from the same participants over time, as well as clustering of different participants living in the same households.

Survival rates were estimated using the Kaplan-Meier method and survival curves were compared using the log-rank test. Nelson-Aalen cumulative hazard functions were also used for visualisation of the cumulative number of expected infections. Multivariable Cox proportional hazards regression models for multiple-record-per-subject data, with time-since-treatment (i.e. 1, 3, 6, 9, or 15 months) as the time-scale and entry into the at-risk period after the treatment, was then used to calculate hazard ratios (HR) and 95% confidence intervals (95% CI) for infection. Covariates included in the Cox model were the age at treatment, sex, SES, and presence/absence of the infection before treatment. Non-significant covariates were removed using backward elimination. A cluster-robust sandwich variance estimator was used to account for the clustering of participants at the household level. All analyses were performed using Stata 16 (StataCorp, College Station, TX, USA). Overall, a *P* < 0.05 was considered as statistically significant.

## Results

### Prevalence of *A. lumbricoides* infection

Summary statistics about *A. lumbricoides* infection prevalence are given in Table 1. The cohort comprised 224 participants living in 55 households, consisting of 44% males and 56% females, with a median age of 13 years (mean 21 years, interquartile range (IQR) 1–72 years). Overall *A. lumbricoides* infection prevalence was 39.7% (95% CI 29.0–51.5%) at baseline. However, over the whole period of sampling, 123 (55%) participants had at least one sample positive for *A. lumbricoides*.

**Table 1.** Prevalence of *A. lumbricoides* in the study population

	*N*^a^	Positives	Prevalence (%)^b^	OR^c^	*P*
Overall	1151	321	27.9 (21.3–35.6)	–	–
Age (pre-treatment)					
≤5 years	226	81	35.8 (23.7–50.2)	Reference	–
6–10 years	262	130	49.6 (38.5–60.8)	1.47 (0.69–3.12)	0.318
11–15 years	157	50	31.8 (22.0–43.7)	0.67 (0.28–1.58)	0.357
16–25 years	131	21	16.0 (8.5–28.2)	0.36 (0.14–0.92)	0.034
26–45 years	226	27	11.9 (7.1–19.3)	0.13 (0.05–0.30)	0.000
≥46 years	149	12	8.1 (3.1–19.3)	0.06 (0.02–0.18)	0.000
Sex					
Males	496	163	32.9 (23.8–43.4)	Reference	–
Females	655	158	24.1 (18.0–31.6)	0.60 (0.37–0.99)	0.047
Sampling					
Pre-treatment	224	89	39.7 (29.0–51.5)	Reference	–
1 month post-treatment	182	14	7.7 (4.3–13.3)	0.03 (0.01–0.06)	0.000
3 months post-treatment	190	37	19.5 (13.2–27.8)	0.14 (0.08–0.27)	0.000
6 months post-treatment	188	61	32.4 (23.1–43.5)	0.46(0.26–0.81)	0.007
9 months post-treatment	177	57	32.2 (23.0–43.0)	0.42(0.24–0.74)	0.003
15 months post-treatment	190	63	33.2 (25.0–42.4)	0.50(0.29–0.88)	0.016
Socio-economic status (Graffar scale)					
Lower-middle (class IV)	452	72	15.9 (9.4–25.8)	Reference	–
Lower (class V)	671	247	36.8 (29.3–45.0)	3.67 (1.27–10.59)	0.016
Unknown	28	2	7.1 (1.9–23.2)	0.71 (0.06–8.55)	0.785

a Total number of samples tested.

b 95% confidence interval within parentheses is adjusted for clustering of participants at the household level.

c OR, Odds ratio, the 95% confidence interval is shown within parentheses. Estimates are adjusted for all variables included in the table. Two nested random effects were included in the model to account for repeated measurements from the same participants over time and clustering of different participants living in the same households.

GLMM analysis revealed that *A. lumbricoides* infection was significantly less prevalent in all age groups above 16 years as compared to children under 5 years of age (16–25 years: Odds Ratio (OR) 0.36, 95% CI 0.14–0.92, *P* = 0.034; 26–45 years: OR 0.13, 95% CI 0.05–0.30, *P* < 0.001; ≥46 years: OR 0.06, 95% CI 0.02–0.18, *P* < 0.001). Prevalence was also significantly lower in females than in males (OR 0.60, 95% CI 0.37–0.99, *P* = 0.047), and at all sampling periods post-treatment as compared to the baseline (1 month: OR 0.03, 95% CI 0.01–0.06, *P* < 0.001; 3 months: OR 0.14, 95% CI 0.08–0.27, *P* < 0.001; 6 months: OR 0.46, 95% CI 0.26–0.81, *P* = 0.007; 9 months: OR 0.42, 95% CI 0.24–0.74, *P* = 0.003; 15 months: OR 0.50, 95% CI 0.29–0.88, *P* = 0.016), but prevalence was significantly higher in people living in households of lower as opposed to lower-middle SES (OR 3.67, 95% CI 1.27–10.59, *P* = 0.016) (Table 1).

Considering the first two samplings (pre-treatment and 1 month post-treatment), 86.2% of the participants that were positive for *A. lumbricoides* before treatment became negative the first month post-treatment.

### Intensity of infection

Table 2 shows the mean intensities of infection (i.e. EPGs) among the positive samples. At baseline, mean EPGs were 30 096 (standard error 5092) for *A. lumbricoides* (89 samples). GLMMs showed that mean EPGs were significantly lower among participants aged 11 years or older as compared to children under 5 years of age (11–15 years: Incidence Rate Ratio (IRR) 0.44, 95% CI 0.24–0.82, *P* = 0.010; 16–25 years: IRR 0.24, 95% CI 0.11–0.52, *P* < 0.001; 26–45 years: IRR 0.18, 95% CI 0.09–0.37, *P* < 0.001; ≥46 years: 0.27, 95% CI 0.09–0.77, *P* = 0.014). Mean EPGs were also significantly lower among samples collected at 1–6 months post-treatment as compared to before treatment (1 month: IRR 0.18, 95% CI 0.09–0.38, *P* < 0.001; 3 months: IRR 0.27, 95% CI 0.16–0.46, *P* < 0.001; 6 months: IRR 0.60, 95% CI 0.38–0.92, *P* = 0.020), but not at 9 or 15 months after treatment. Moreover, mean EPGs were significantly higher in people living in households of lower compared to lower-middle SES (IRR 1.87, 95% CI 1.03–3.38, *P* = 0.039) (Table 2).

**Table 2.** Mean intensity of infection (eggs per gram of faeces – EPG) for *A. lumbricoides* in the study population

	*N*^a^	Mean intensity (EPG)^b^	IRR^c^	*P*
Overall	321	26 167 (20 868–31 466)	–	–
Age (pre-treatment)				
≤5 years	81	29 022 (22 511–35 532)	Reference	–
6–10 years	130	36 233 (27 899–44 567)	1.03 (0.63–1.74)	0.853
11–15 years	50	16 446 (10 183–22 709)	0.44 (0.24–0.82)	0.010
16–25 years	21	9423 (2643–16 203)	0.24 (0.11–0.52)	0.000
26–45 years	27	7759 (3365–12 147)	0.18 (0.09–0.37)	0.000
≥46 years	12	9081 (3–18 159)	0.27 (0.09–0.77)	0.014
Sex				
Males	163	24 748 (17 341–32 155)	Reference	–
Females	158	27 631 (20 317–34 946)	0.97 (0.67–1.41)	0.884
Sampling				
Pre-treatment	89	30 096 (19 538–40 653)	Reference	–
1 month post-treatment	14	10 659 (842–20 476)	0.18 (0.09–0.38)	0.000
3 months post-treatment	37	16 368 (2194–30 541)	0.27 (0.16–0.46)	0.000
6 months post-treatment	61	25 147 (17 595–32 699)	0.60 (0.38–0.92)	0.020
9 months post-treatment	57	25 583 (14 860–36 306)	0.70 (0.44–1.09)	0.111
15 months post-treatment	63	31 336 (17 389–45 283)	1.02 (0.65–1.59)	0.940
Socio-economic status (Graffar scale)				
Lower-middle (class IV)	72	23 996 (10 700–37 292)	Reference	
Lower (class V)	247	26 991 (21 359–32 623)	1.87 (1.03–3.38)	0.039
Unknown	2	2511 (1536–3487)	0.33 (0.04–2.54)	0.287

a Total number of positive samples.

b Calculated based on positive stool samples only, 95% confidence interval within parentheses adjusted for clustering of participants at the household level.

c IRR, Incidence rate ratio, the 95% confidence interval is shown within parentheses. Estimates are adjusted for all variables included in the table and for clustering of participants at the household level. Two nested random effects were included in the model to account for repeated measurements from the same participants over time and clustering of different participants living in the same households.

### Survival analysis

**Fig. 1.**
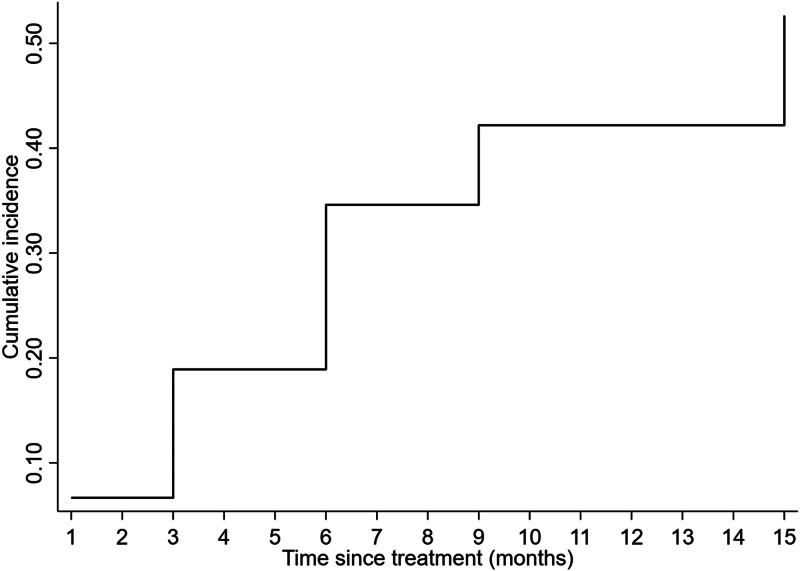
Nelson-Aalen cumulative incidence of *A. lumbricoides* infections over time since anthelmintic treatment.

**Fig. 2.**
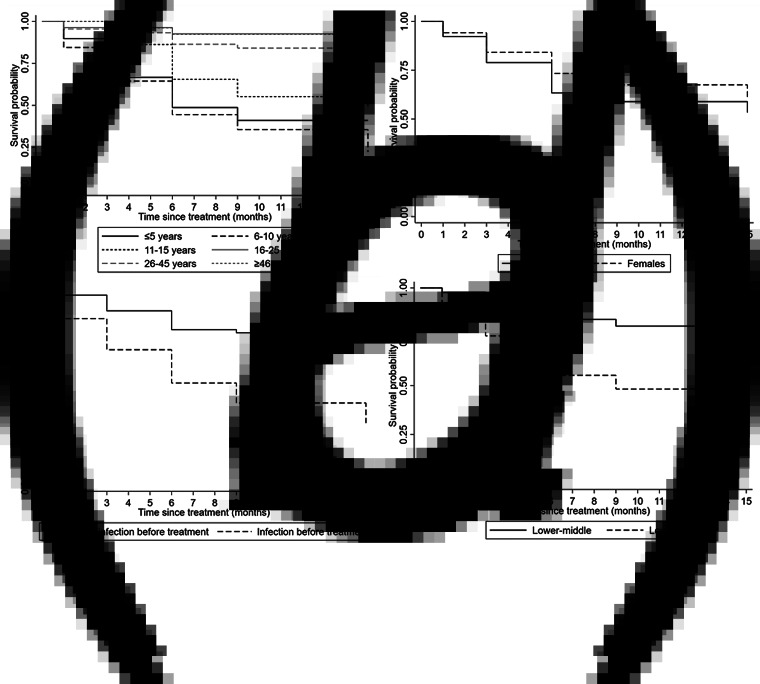
Kaplan-Meier survival curves for *A. lumbricoides* infections over time since anthelmintic treatment according to age (a), gender (b), previous infection before treatment (c) and socio-economic status (d).

Survival analysis for the time to (re)infection after treatment was based on 210 participants that received treatment (i.e. 14 participants could not be treated because they were absent from home at the time of treatment and were therefore excluded from further analyses). The included participants accounted for a total of 2357 person-months at risk during which 90 infections were observed, resulting in an overall incidence of 3.8 *A. lumbricoides* infections per 100 person-months (95% CI 3.1–4.7). Median time from treatment to infection was 6 months (mean 6 months, IQR 3–9): at 1, 3, 6, 9 and 15 months post-treatment, the cumulative incidence of *A. lumbricoides* infection was 6.7%, 18.9%, 34.6%, 42.2%, and 52.6%, respectively (Fig. 1). The median age at *A. lumbricoides* infection after treatment was 8 years (mean 13 years, IQR 5–13 years). Survival curves for *A. lumbricoides* infection differed significantly depending on age, SES, and prior (i.e. pre-treatment) *A. lumbricoides* infection (Fig. 2), but not sex. The Cox proportional hazards model for *A. lumbricoides* infection (Table 3) also showed that the HRs in all age groups ≥16 years were significantly lower than in children ≤5 years. Moreover, those who had an *A. lumbricoides* infection before treatment (compared to those who did not have this infection before treatment) were at increased risk of being (still or again) positive for *A. lumbricoides* after the treatment. The same was true for people living in households of lower SES. No significant differences were observed for sex (Table 3).

**Table 3.** Significant results of the Cox proportional hazards regression model for *A. lumbricoides* (re)infection after treatment.

	*N*^a^	Obs^b^	Exp^c^	Person-months at risk	HR (95% CI)^d^	*P*
Overall	210	90	–	2357	–	–
Age (before treatment)						
≤5 years	39	23	14.1	340	Reference	–
6–10 years	45	35	15.4	364	1.13 (0.72–1.72)	0.589
11–15 years	29	15	12.5	315	0.65 (0.33–1.26)	0.201
16–25 years	27	4	13.6	382	0.17 (0.05–0.56)	0.003
26–45 years	44	9	12.3	587	0.29 (0.14–0.59)	0.001
≥46 years	26	4	13.2	369	0.20 (0.07–0.52)	0.001
*A. lumbricoides* infection before treatment						
No	123	31	58.1	1577	Reference	–
Yes	87	59	31.9	780	1.94 (1.19–3.17)	0.008
Socio-economic status (Graffar scale)						
Lower-middle (class IV)	87	26	41.1	1127	Reference	–
Lower (class V)	114	64	44.1	1095	1.92 (1.13–3.27)	0.016
Unknown	9	0	4.7	135	–	–

a Number of participants that received treatment with observations not beginning on or after infection.

b Observed number of *A. lumbricoides* infections.

c Expected number of *A. lumbricoides* infections.

d HR, hazard ratio, 95% CI, 95% confidence interval. Estimates are adjusted for the variables included in the table and for clustering of participants at the household level; months after treatment is the underlying time scale.

## Discussion

In this study, effects of age, sex, SES and pre-treatment infection status were observed on the risk of *A. lumbricoides* (re)infection after treatment. Older age appeared to be a protective factor for *A. lumbricoides* in terms of both occurrence and intensity of infection. A reduced prevalence, but not intensity of infection, was observed in females compared to males. Increased prevalence and intensity of infection were found to be associated with lower SES. Moreover, the rate of *A. lumbricoides* (re)infection after treatment was higher within younger age groups, lower SES and presence of prior *A. lumbricoides* infection.

Age is perhaps the most common predictor of STHs in general and defines clear target groups with the highest parasitic load in an endemically infected community. Typically, the age-related intensity of infection in most settings shows a peak at 5–10 years of age for *A. lumbricoides* [27, 28]. Also in this study, *A. lumbricoides* prevalence peaked in children of 6–10 years of age, with significantly lower prevalence in older age groups. Furthermore, the intensity of infection (and hence the capacity of polluting soil with parasite eggs), was significantly higher in <10 year old children, which accounted for 60% of the total egg output in the community. These findings suggest that *A. lumbricoides* control programmes based on mass drug administration would be more effective when targeting children of 2–12 years of age in resource-limited settings, as suggested by the WHO Roadmap for STH control (9).

Regarding sex differences in STH prevalence, a meta-analysis has shown a significantly higher prevalence of *A. lumbricoides* in females [29]. However, in this study, we detected a significantly lower prevalence in females than males. These results may be a reflection of lower exposure to the parasites among females in the community, as males in this rural community are typically more likely to engage in leisure or agricultural activities that bring them to closer and more frequent contacts with soil. Nevertheless, sex difference was not found for parasitic loads.

SES has often been advocated as a proxy for the high prevalence and intensity of infection for STHs. Indeed, several studies have found a relation between poverty, poor housing conditions, overcrowding, lack of basic services, and means of faecal disposal, and have been associated with spatial clustering of infections in certain households [19, 27, 30–33]. In the current study, some of these factors were found to be significantly associated with the participant's ‘worminess’ [27]. The community under study has seen a decline in living conditions in the past 15 years. Indeed, the descendants of the founder population had reasonable State-built rural housing in the 1980s, but they faced severe deterioration of living conditions later on (e.g. irregular indoor water supply, and consequently no or little use of toilets) as a result of the political and socio-economic crisis affecting the country. Furthermore, the subsequent generation of community residents has had little opportunity to access proper housing and sanitisation, but rather live in self-build houses made of waste material with virtually no sanitation. It is therefore conceivable that several of such SES-related components measured by the Graffar system acted as a proxy for the risk of *A. lumbricoides* infection in our study.

Pyrantel is usually highly effective against *A. lumbricoides* at a single dose [17], as also shown in this study. Similar to findings reported elsewhere [19, 21], the community prevalence returned to almost pre-treatment levels at six months post-treatment. The intensity of infection showed a similar pattern, returning to pre-treatment levels at nine months after treatment. These results, when compared to those obtained with albendazole or mebendazole treatment [17, 34], question the efficacy of annual or even biannual mass drug administrations for *A. lumbricoides* [14]. Therefore, it may be more effective, albeit more expensive, to carry out three consecutive days treatment regimens, repeated at least twice a year for a mid-term programme of five years, and to cover also STHs other than *A. lumbricoides*, such as *Trichuris trichiura* and hookworms [18–21], which are other two STHs included in the WHO Roadmap for Neglected Tropical Diseases [9], thereby including enough time to develop the means of sanitation [34, 35].

Survival analysis showed that the rate of (re)acquisition of *A. lumbricoides* infection after treatment was slower with increasing age. Indeed, by the end of the 15-month study period, the probability of being infection-free among children was below 50%, whereas it remained above 75% among adults. Similar age effects have been described before [21, 28], and are related to both immunity and increased exposure to *A. lumbricoides* among children. Yet, the strongest predictor was having had an *A. lumbricoides* infection before the treatment, suggesting an intrinsic proneness to *A. lumbricoides* infection in some individuals, the so-called ‘wormy’ persons. These findings support the importance of young age in defining the targets of mass drug administration programmes, but on the other hand, they highlight that STHs tend to cluster and recur in some people [27, 29]. The reasons as to why some individuals tend to acquire the infection more frequently than others can be both epidemiological (increased exposure to the parasite, poor hygiene, etc.) and biological (e.g. increased susceptibility to infection), and these persons are the largest contributors to the environmental pollution with worm eggs [19, 21, 27, 29, 36–38]. Household clustering of wormy persons has been described before [15, 16, 27, 29], highlighting the potential of targeted control efforts in rural communities. However, it is also true that given the pre-patent period of *A. lumbricoides*, some of the infections observed one month after treatment might be persistent infections that were not cleared by the treatment rather than new infections. Regardless, their impact can be generally expected to be limited, as the large majority (86.2%) of the participants that were positive before the treatment were found to be negative one month after the treatment, meaning that ≤13.8% of them may be persistent infections contributing to the relatively low community prevalence observed at one month post-treatment. While this study cannot fully discern whether these were new or persistent infections, the analyses included the pre-treatment infection status of the participants as a variable of interest in order to take into account the effect of having had the infection previously. Other factors that might influence the pre-patent period like age were also taken into account. Moreover, although the presence of possible persistent infections needs to be considered in the interpretation of some results, it also offers the opportunity to observe the natural rebuilding of the community levels of *A. lumbricoides* infection in a real-world setting where inevitably an imperfect treatment could be applied. Cross-infection of humans with *Ascaris suum* from pigs cannot be excluded as well. Indeed, there is evidence indicating that cross-infection may occur under certain circumstances, particularly in industrialised countries where *A. lumbricoides* occurs very rarely, but pig infection with *A. suum* is common. Humans have therefore been found to be infected and molecular markers have shown that the worms responsible for such infection are genetically close to *A. suum* [39]. Pigs bred domestically, with basically no biosecurity measures in place, are common in the sampled community. Consequently, it cannot be excluded that cross-infection of humans with *A. suum* occurred.

A final consideration pertains to the time elapsed between data collection and analysis (11 years). However, because of the protracted socioeconomic and political crisis in the country, it is unlikely that the dynamics of STHs have changed in the study area, as no improvements of the country and local living conditions have occurred ever since [40–43]. Moreover, our observations might be generalisable to similar situations in other resource-limited settings worldwide and provide historical data for comparison with other studies as well.

In conclusion, younger age, lower SES and pre-treatment infection status are significant predictors of *A. lumbricoides* (re)infection after treatment, the burden of which seems to regain its community baseline levels after six months. Our findings seem to suggest that mass drug administration protocols would benefit from considering these factors in selective treatment strategies and possibly more than just annual or biannual treatments in the target population.

## Data Availability

All data relevant to the study are included in the article in an aggregated and anonymised format. For legal reasons, the disaggregated dataset is available in an anonymised format from the first author on reasonable request.
